# Tension Pneumothorax, Pneumoperitoneum, and Cervical Emphysema following a Diagnostic Colonoscopy

**DOI:** 10.1155/2013/583287

**Published:** 2013-05-30

**Authors:** Ali Pourmand, Hamid Shokoohi

**Affiliations:** Department of Emergency Medicine, George Washington University, Washington, DC 20037, USA

## Abstract

Colonoscopy is currently a widespread procedure used in screening for colorectal cancer. Iatrogenic colonic perforation during colonoscopy is a serious and potentially life-threatening complication that can cause significant morbidity and mortality. “Triple pneumo” (a combination of pneumothorax, pneumomediastinum, and pneumoperitoneum) following colonoscopy is a rare but a serious condition requiring immediate diagnosis and emergent intervention. In majority of these cases a colonic perforation is the initial injury that is followed by pneumothorax and pneumomediastinum through the potential anatomical connection with retroperitoneal and mediastinal spaces. In this rare case report we are presenting a case of “triple pneumo” with no evidence of colonic perforation. This patient developed a simultaneous pneumoperitoneum, pneumomediastinum, and a tension pneumothorax requiring immediate tube thoracostomy. This case may raise the awareness on the likelihood of these serious complications after colonoscopy.

## 1. Introduction 

Colonoscopy has been used as a safe diagnostic method in gastroenterology for the last four decades [1]. Routine screening for colorectal cancer and evaluating high risk patients with family history of colorectal polyps or colon cancer are among the most common indications to perform a diagnostic colonoscopy [2–5]. Gastrointestinal perforation is one of the most serious and potentially life-threatening complications of colonoscopy that may result in a combination of pneumoperitoneum, pneumothoraces, and pneumomediastinum (“triple pneumo”). In this report, we present a rare case of tension pneumothorax with a simultaneous pneumoperitoneum and subcutaneous emphysema following a diagnostic colonoscopy who presented to the emergency department (ED) in a critical condition. The purpose of this report is to raise the awareness on the likelihood of these complications after colonoscopy.

## 2. Case Report

The patient was an 84-year-old woman who was presented to the ED by emergency medical services (EMS), with acute onset of abdominal pain, changes in mental status, and tachycardia after outpatient diagnostic colonoscopy. Upon completion of the procedure, the patient reported that she had abdominal pain, chest pain, and shortness of breath. The physician's reevaluation at that time revealed an altered mental status and persistent tachycardia. On EMS arrival, patient was tachypneic with a respiratory rate of 32, and hypoxic with an oxygen saturation of 88% on room air. The patient was placed on oxygen mask and transported to the ED. On ED arrival, patient was awake but confused, complaining of abdominal pain, chest pain, and shortness of breath. The patient had a blood pressure of 105/60, a heart rate of 125, and an oxygen saturation of 91% on a 100% non-rebreather. The patient's airway was intact and she was able to talk. Auscultation of the lungs revealed no breath sound on the right hemithorax but a normal breath sound on the left side. Cardiac exam revealed tachycardia and normal S1, and S2, with no murmur. Abdominal exam demonstrated a soft abdomen, with a mild, suprapubic tenderness, no distension, and without peritoneal sign. 

**Figure 1:**
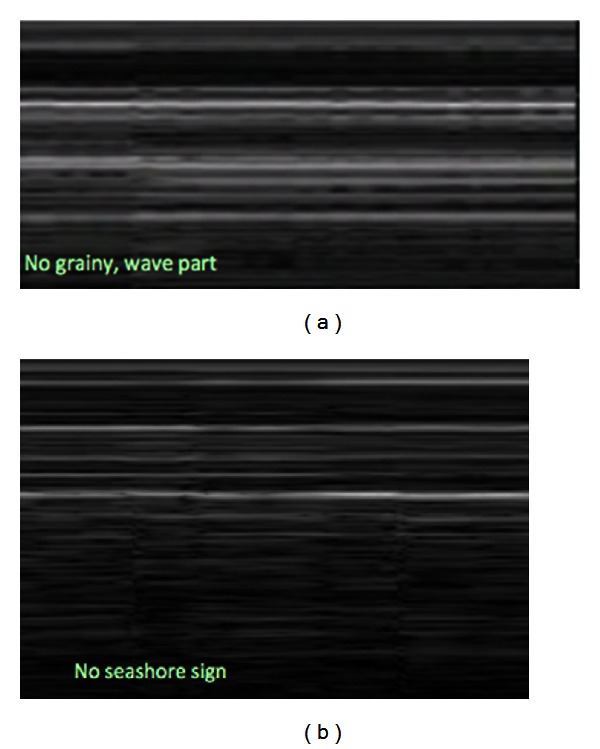
Pneumothorax diagnosed using ultrasound.

**Figure 2:**
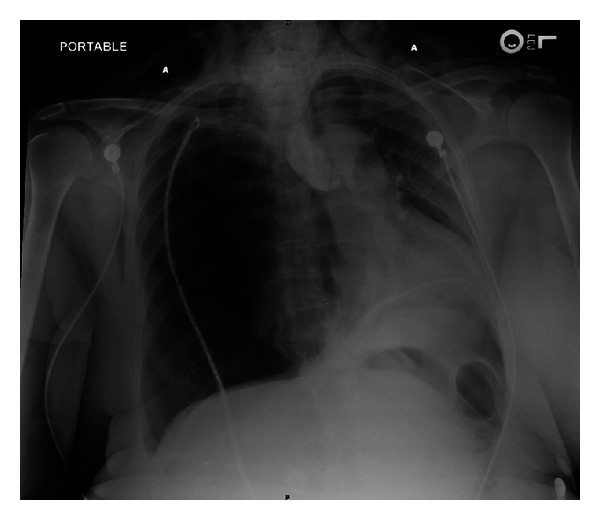
Tension pneumothorax. (a) Cervical emphysema.

A bedside ultrasound of the anterior chest was performed using the Sonosite MicroMaxx system (Sonosite, Bothell, WA, USA) and a 13–6 MHz linear array transducer. The ultrasound findings included the lack of pleural sliding sign and a barcode sign in M-Mode, indicating a possible pneumothorax (Figure 1). Chest X-ray revealed a tension pneumothorax with a significant left sided cardiac shift, and no evidence of intestinal structures in the chest (Figure 2). Chest X-ray and diagnostic ultrasound, performed simultaneously at the same time. Palpation of the neck and anterior chest revealed subcutaneous emphysema. Shortly after ED arrival, blood pressure dropped to 95/50, and percutaneous needle decompression was performed by using a 14 G catheter, followed by a tube thoracostomy placement to the right side. The patient was stable with a blood pressure of 145/92 and a heart rate of 95 and underwent a CT scan of the abdomen, which revealed the presence of a pneumoperitoneum. After consulting with general surgery and the cardiothoracic surgery team, the patient was admitted to the critical care unit for observation. The hospital clinical course was uneventful with the patient in a stable hemodynamic state. The patient was discharged home a week later in good condition. 

## 3. Discussion

Iatrogenic colonic perforation following colonoscopy is a rare but serious complication. Dafnis et al. reported that the overall morbidity for 6066 patients that underwent colonoscopy was 0.4%, and specifically 0.2% for diagnostic procedures and 1.2% for therapeutic procedures [6]. The most frequent complications were bleeding, accounting for 0.2%, mainly with diagnostic procedures, and perforation accounting for 0.1%, mainly with therapeutic intervention [6]. Tulchinsky et al. observed colonoscopic complications resulting in morbidity to be as low as 0.058% over an 8-years study [7]. Despite the low morbidity rate, there can be serious life-threatening complications such as pulmonary emboli, congestive heart failure, sepsis, and death [8–10]. They identified three methods as potential causes of colonic perforation during colonoscopy: barotrauma, mechanical related trauma, and therapeutic associated trauma [11]. 

In our case rectal contrast outlined the colon and did not show any perforation. There is consistent data showing that approximately 85% of visceral perforations present with pneumoperitoneum [12, 13]. Interestingly, pneumoperitoneum can present without any visceral perforation in about 5 to 15% of cases and requires nonsurgical intervention [12–14]. Mularski et al. presented eight cases of pneumoperitoneum, two of which had a negative laparotomy, and six of which were managed nonsurgically [14]. We interviewed our patient after a negative CT scan for perforation, and the patient reported that she had 2 rectal surgeries in 1949 and 1951 for hemorrhoidectomy. This procedure could explain the air leakage from the rectal anastomoses. Pneumoperitoneum can be caused by air entering the retroperitoneal space, directly leaking from the rectal anastomoses. In our case, the air leakage from the diagnostic procedure was complicated by a tension pneumothorax. The anatomical connection between retroperitoneal and mediastinal spaces could describe this complication. Maunder et al. described the anatomical connections between the cervical area, the mediastinum, and the retroperitoneum [15]. The visceral space starts from the cervical area in the anterior midline and extends to the upper mediastinum. This space contains the larynx, thyroid gland, cervical esophagus, and cervical trachea. The visceral space then continues, surrounding esophagus as it enters into the mediastinum and then through the diaphragmatic hiatus into the abdomen [15, 16]. This continuity describes the mechanism of air entry from the retroperitoneal region into the mediastinum and the region of the neck. In our case, patient underwent a close observation and conservative nonsurgical management, with an uneventful hospital course.

## 4. Conclusion

A combination of tension pneumothorax, pneumomediastinum, and pneumoperitoneum following colonoscopy is a rare but potentially serious condition, particularly in frail elderly patients with multiple comorbidities. In this case report we presented an elderly patient who developed an iatrogenic tension pneumothorax and pneumoperitoneum in critical condition. We were able to attain a correct diagnosis using bedside ultrasound and a CT scan. It is critical that physicians be aware of these complications in order to facilitate early recognition and treatment in efforts to optimize patient outcome.
